# 1*H*-1,2,4-Triazol-4-ium (3,4-dichloro­phen­yl)methane­sulfonate

**DOI:** 10.1107/S1600536811053062

**Published:** 2011-12-14

**Authors:** Ling Zhang, Guri L. V. Damu, Jing-Song Lv, Rong-Xia Geng, Cheng-He Zhou

**Affiliations:** a Laboratory of Bioorganic & Medicinal Chemistry, School of Chemistry and Chemical Engineering, Southwest University, Chongqing 400715, People’s Republic of China

## Abstract

In the title molecular salt, C_2_H_4_N_3_^+^·C_7_H_5_Cl_2_O_3_S^−^, C—C—S angle [112.25 (18)°] deviates slightly from that expected for ideal *sp* ^3^-hybridization geometry. In the crystal, the components are linked by N—H⋯O and bifurcated N—H⋯(O,O) hydrogen bonds into chains parallel to [110].

## Related literature

For applications of triazole compounds, see: Sen *et al.* (2010 ▶); Subbaraman *et al.* (2009 ▶); Wang & Zhou (2011 ▶); Zhou *et al.* (2009 ▶); Bai *et al.* (2007 ▶); Chang *et al.*(2011 ▶). 
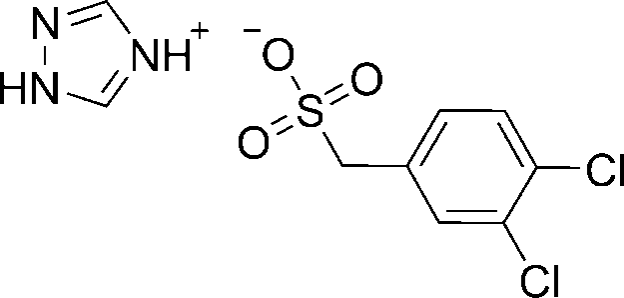


## Experimental

### 

#### Crystal data

C_2_H_4_N_3_^+^·C_7_H_5_Cl_2_O_3_S^−^
*M* *_r_* = 310.15
Triclinic, 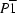

*a* = 5.2430 (6) Å
*b* = 8.2970 (8) Å
*c* = 14.5656 (15) Å
α = 94.330 (5)°
β = 98.387 (6)°
γ = 92.292 (5)°
*V* = 624.22 (11) Å^3^
*Z* = 2
Mo *K*α radiation
μ = 0.69 mm^−1^
*T* = 296 K
0.30 × 0.28 × 0.25 mm

#### Data collection

Bruker SMART CCD diffractometer
Absorption correction: multi-scan (*SADABS*; Sheldrick, 1996 ▶) *T*_min_ = 0.820, *T*_max_ = 0.846
8971 measured reflections
2196 independent reflections
2000 reflections with *I* > 2σ(*I*)
*R*_int_ = 0.030

#### Refinement

*R*[*F* ^2^ > 2σ(*F* ^2^)] = 0.044
*wR*(*F* ^2^) = 0.124
*S* = 1.03
2196 reflections
180 parameters
H atoms treated by a mixture of independent and constrained refinement
Δρ_max_ = 0.69 e Å^−3^
Δρ_min_ = −0.60 e Å^−3^

### 

Data collection: *SMART* (Bruker, 2001 ▶); cell refinement: *SAINT* (Bruker, 2001 ▶); data reduction: *SAINT*; program(s) used to solve structure: *SHELXS97* (Sheldrick, 2008 ▶); program(s) used to refine structure: *SHELXL97* (Sheldrick, 2008 ▶); molecular graphics: *PLATON* (Spek, 2009 ▶); software used to prepare material for publication: *SHELXTL* (Sheldrick, 2008 ▶).

## Supplementary Material

Crystal structure: contains datablock(s) global, I. DOI: 10.1107/S1600536811053062/lh5392sup1.cif

Structure factors: contains datablock(s) I. DOI: 10.1107/S1600536811053062/lh5392Isup2.hkl

Supplementary material file. DOI: 10.1107/S1600536811053062/lh5392Isup3.cml

Additional supplementary materials:  crystallographic information; 3D view; checkCIF report

## Figures and Tables

**Table 1:** Hydrogen-bond geometry (Å, °)

*D*—H⋯*A*	*D*—H	H⋯*A*	*D*⋯*A*	*D*—H⋯*A*
N1—H1*M*⋯O1^i^	0.85 (4)	1.96 (4)	2.709 (3)	146 (4)
N3—H4*M*⋯O2^ii^	0.79 (4)	2.08 (4)	2.768 (3)	146 (3)
N3—H4*M*⋯O2^iii^	0.79 (4)	2.54 (3)	3.089 (3)	128 (3)

Symmetry codes: (i) 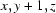; (ii) 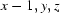; (iii) 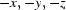.

**Fig. 1.**
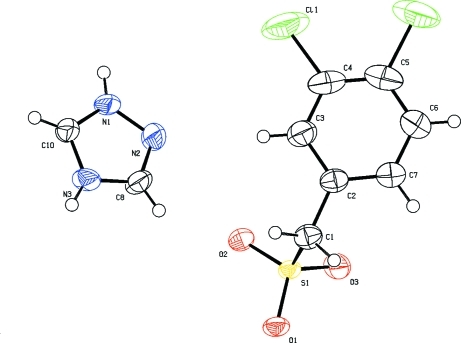
The molecular structure of (I) showing displacement ellipsoids drawn at the 50% probability level.

## supplementary crystallographic information

### Comment

Triazole is a unique molecule which could exert diverse non-covalent interactions and endow triazole derivatives to exhibit various potential appplications in medicinal chemistry (Wang & Zhou, 2011), argrochemical and chemical fields (Bai *et al.*, 2007) and material science (Chang *et al.*, 2011). Work has shown that triazole derivatives could be used as proton transport facilitators for sulfonic acid based membranes for high temperature fuel cell operations (Sen *et al.*, 2010; Subbaraman *et al.*, 2009). Our interest is to investigate the intreractions of triazole compounds with diverse anions for the formation of supramolecular drugs (Zhou *et al.*, 2009). Herein we report the crystal structure of title compound.

In the molecular structure the title compound (Fig. 1) there is a slight deviation of the C2—C1—S1 angle (112.25°) in terms ideal sp^3^ hybridization geometry. In the crystal, the components are linked by N—H···O hydrogen bonds and bifurcated N—H···(O,O) into one dimensional chains along [110].

### Experimental

A crystal of title the compound suitable for X-ray analysis was grown from the solution of 1,2,4-triazole and (3,4-dichlorophenyl)methanesulfonic acid in methanol by slow evaporation at room temperature.

### Refinement

The H atoms of the anion were placed in calculated positions with C—H = 0.93Å (aromatic) and 0.97Å (methylene) and refined in a riding-motion approximation with U_iso_(H) = 1.2U_eq_(C). All H atoms in the cation were refined independently with isotropic displacement parameters.

### Crystal data

**Table:**

C_2_H_4_N_3_^+^·C_7_H_5_Cl_2_O_3_S^−^	*Z* = 2
*M**_r_* = 310.15	*F*(000) = 316
Triclinic, *P*1	*D*_x_ = 1.650 Mg m^−^^3^
Hall symbol: -P 1	Mo *K*α radiation, λ = 0.71073 Å
*a* = 5.2430 (6) Å	Cell parameters from 4951 reflections
*b* = 8.2970 (8) Å	θ = 2.8–27.5°
*c* = 14.5656 (15) Å	µ = 0.69 mm^−^^1^
α = 94.330 (5)°	*T* = 296 K
β = 98.387 (6)°	Block, colorless
γ = 92.292 (5)°	0.30 × 0.28 × 0.25 mm
*V* = 624.22 (11) Å^3^	

### Data collection

**Table:**

Bruker SMART CCD diffractometer	2196 independent reflections
Radiation source: fine-focus sealed tube	2000 reflections with *I* > 2σ(*I*)
graphite	*R*_int_ = 0.030
φ scans and ω scans with κ offsets	θ_max_ = 25.0°, θ_min_ = 2.5°
Absorption correction: multi-scan (*SADABS*; Sheldrick, 1996)	*h* = −6→6
*T*_min_ = 0.820, *T*_max_ = 0.846	*k* = −9→9
8971 measured reflections	*l* = −17→17

### Refinement

**Table:**

Refinement on *F*^2^	Secondary atom site location: difference Fourier map
Least-squares matrix: full	Hydrogen site location: inferred from neighbouring sites
*R*[*F*^2^ > 2σ(*F*^2^)] = 0.044	H atoms treated by a mixture of independent and constrained refinement
*wR*(*F*^2^) = 0.124	*w* = 1/[σ^2^(*F*_o_^2^) + (0.0656*P*)^2^ + 0.6305*P*] where *P* = (*F*_o_^2^ + 2*F*_c_^2^)/3
*S* = 1.03	(Δ/σ)_max_ < 0.001
2196 reflections	Δρ_max_ = 0.69 e Å^−^^3^
180 parameters	Δρ_min_ = −0.60 e Å^−^^3^
0 restraints	Extinction correction: *SHELXL97* (Sheldrick, 2008), Fc^*^=kFc[1+0.001xFc^2^λ^3^/sin(2θ)]^-1/4^
Primary atom site location: structure-invariant direct methods	Extinction coefficient: 0.064 (7)

### Special details

**Table:**

Geometry. All e.s.d.'s (except the e.s.d. in the dihedral angle between two l.s. planes) are estimated using the full covariance matrix. The cell e.s.d.'s are taken into account individually in the estimation of e.s.d.'s in distances, angles and torsion angles; correlations between e.s.d.'s in cell parameters are only used when they are defined by crystal symmetry. An approximate (isotropic) treatment of cell e.s.d.'s is used for estimating e.s.d.'s involving l.s. planes.
Refinement. Refinement of *F*^2^ against ALL reflections. The weighted *R*-factor *wR* and goodness of fit *S* are based on *F*^2^, conventional *R*-factors *R* are based on *F*, with *F* set to zero for negative *F*^2^. The threshold expression of *F*^2^ > σ(*F*^2^) is used only for calculating *R*-factors(gt) *etc*. and is not relevant to the choice of reflections for refinement. *R*-factors based on *F*^2^ are statistically about twice as large as those based on *F*, and *R*- factors based on ALL data will be even larger.

### Fractional atomic coordinates and isotropic or equivalent isotropic displacement parameters (Å^2^)

**Table:**

	*x*	*y*	*z*	*U*_iso_*/*U*_eq_	
H4M	−0.257 (7)	0.126 (5)	0.067 (2)	0.059 (10)*	
C1	0.5971 (5)	−0.1996 (3)	0.24949 (19)	0.0405 (6)	
H1A	0.4222	−0.1654	0.2491	0.049*	
H1B	0.6118	−0.2993	0.2801	0.049*	
C2	0.7822 (5)	−0.0722 (3)	0.30368 (18)	0.0393 (6)	
C3	0.7467 (7)	0.0915 (4)	0.2965 (2)	0.0522 (8)	
H3	0.6021	0.1240	0.2589	0.063*	
C4	0.9246 (8)	0.2072 (4)	0.3449 (2)	0.0577 (9)	
C5	1.1357 (8)	0.1603 (4)	0.4029 (2)	0.0634 (9)	
C6	1.1715 (8)	−0.0016 (5)	0.4095 (3)	0.0751 (11)	
H6	1.3146	−0.0343	0.4478	0.090*	
C7	0.9978 (7)	−0.1156 (4)	0.3600 (2)	0.0560 (8)	
H7	1.0266	−0.2248	0.3647	0.067*	
C8	0.1007 (6)	0.1330 (3)	0.1058 (2)	0.0448 (7)	
C10	−0.1080 (5)	0.3440 (3)	0.0826 (2)	0.0404 (6)	
Cl1	0.8778 (3)	0.40861 (12)	0.33114 (10)	0.1080 (5)	
Cl2	1.3556 (3)	0.30086 (16)	0.46816 (10)	0.1064 (5)	
N1	0.1333 (4)	0.3833 (3)	0.10869 (16)	0.0393 (6)	
N2	0.2733 (4)	0.2507 (3)	0.12354 (19)	0.0476 (6)	
N3	−0.1339 (4)	0.1855 (3)	0.08066 (18)	0.0423 (6)	
O1	0.4994 (3)	−0.3828 (2)	0.09619 (13)	0.0383 (5)	
O2	0.5688 (4)	−0.0964 (2)	0.08476 (13)	0.0417 (5)	
O3	0.9286 (3)	−0.2589 (2)	0.13509 (14)	0.0446 (5)	
S1	0.65640 (11)	−0.23697 (7)	0.13202 (4)	0.0291 (2)	
H3M	0.132 (7)	0.035 (5)	0.108 (3)	0.070 (11)*	
H2M	−0.232 (8)	0.408 (5)	0.070 (3)	0.069 (11)*	
H1M	0.197 (8)	0.480 (5)	0.111 (3)	0.069 (11)*	

### Atomic displacement parameters (Å^2^)

**Table:**

	*U*^11^	*U*^22^	*U*^33^	*U*^12^	*U*^13^	*U*^23^
C1	0.0418 (15)	0.0362 (14)	0.0448 (15)	−0.0018 (11)	0.0112 (12)	0.0037 (11)
C2	0.0474 (16)	0.0346 (14)	0.0369 (13)	0.0005 (11)	0.0119 (12)	0.0001 (11)
C3	0.067 (2)	0.0388 (16)	0.0495 (16)	0.0047 (14)	0.0037 (14)	0.0031 (13)
C4	0.089 (3)	0.0335 (16)	0.0525 (18)	−0.0047 (16)	0.0240 (18)	−0.0040 (13)
C5	0.069 (2)	0.057 (2)	0.060 (2)	−0.0170 (17)	0.0137 (17)	−0.0196 (16)
C6	0.069 (2)	0.061 (2)	0.083 (3)	0.0053 (18)	−0.020 (2)	−0.0160 (19)
C7	0.060 (2)	0.0419 (17)	0.0606 (19)	0.0051 (14)	−0.0021 (15)	−0.0072 (14)
C8	0.0402 (16)	0.0259 (14)	0.0685 (19)	0.0036 (11)	0.0082 (13)	0.0052 (13)
C10	0.0316 (14)	0.0324 (14)	0.0553 (16)	0.0028 (11)	0.0013 (12)	0.0011 (12)
Cl1	0.1756 (14)	0.0328 (5)	0.1118 (9)	−0.0042 (6)	0.0125 (9)	0.0028 (5)
Cl2	0.1059 (10)	0.0813 (8)	0.1162 (10)	−0.0352 (7)	−0.0009 (7)	−0.0397 (7)
N1	0.0373 (13)	0.0243 (12)	0.0543 (14)	−0.0071 (9)	0.0030 (10)	0.0023 (10)
N2	0.0283 (12)	0.0420 (14)	0.0704 (16)	0.0012 (10)	0.0000 (11)	0.0059 (12)
N3	0.0267 (12)	0.0332 (12)	0.0651 (16)	−0.0091 (10)	0.0086 (11)	−0.0048 (11)
O1	0.0332 (10)	0.0229 (9)	0.0571 (11)	−0.0051 (7)	0.0052 (8)	−0.0026 (8)
O2	0.0462 (11)	0.0250 (9)	0.0518 (11)	−0.0040 (8)	−0.0012 (8)	0.0092 (8)
O3	0.0264 (10)	0.0443 (11)	0.0617 (12)	−0.0004 (8)	0.0071 (8)	−0.0051 (9)
S1	0.0251 (4)	0.0196 (3)	0.0417 (4)	−0.0026 (2)	0.0042 (2)	0.0009 (2)

### Geometric parameters (Å, °)

**Table:**

C1—C2	1.500 (4)	C7—H7	0.9300
C1—S1	1.790 (3)	C8—N2	1.289 (4)
C1—H1A	0.9700	C8—N3	1.332 (4)
C1—H1B	0.9700	C8—H3M	0.84 (4)
C2—C7	1.374 (4)	C10—N1	1.288 (4)
C2—C3	1.388 (4)	C10—N3	1.314 (4)
C3—C4	1.387 (5)	C10—H2M	0.86 (4)
C3—H3	0.9300	N1—N2	1.360 (3)
C4—C5	1.378 (6)	N1—H1M	0.86 (4)
C4—Cl1	1.721 (3)	N3—H4M	0.79 (4)
C5—C6	1.373 (6)	O1—S1	1.4538 (17)
C5—Cl2	1.732 (3)	O2—S1	1.4550 (19)
C6—C7	1.371 (5)	O3—S1	1.4405 (19)
C6—H6	0.9300		
			
C2—C1—S1	112.25 (18)	C6—C7—H7	119.3
C2—C1—H1A	109.2	C2—C7—H7	119.3
S1—C1—H1A	109.2	N2—C8—N3	111.8 (3)
C2—C1—H1B	109.2	N2—C8—H3M	124 (3)
S1—C1—H1B	109.2	N3—C8—H3M	124 (3)
H1A—C1—H1B	107.9	N1—C10—N3	106.9 (3)
C7—C2—C3	118.1 (3)	N1—C10—H2M	128 (3)
C7—C2—C1	120.3 (3)	N3—C10—H2M	126 (3)
C3—C2—C1	121.6 (3)	C10—N1—N2	111.6 (2)
C4—C3—C2	120.7 (3)	C10—N1—H1M	123 (3)
C4—C3—H3	119.7	N2—N1—H1M	125 (3)
C2—C3—H3	119.7	C8—N2—N1	103.0 (2)
C5—C4—C3	120.0 (3)	C10—N3—C8	106.8 (2)
C5—C4—Cl1	120.9 (3)	C10—N3—H4M	131 (3)
C3—C4—Cl1	119.0 (3)	C8—N3—H4M	122 (3)
C6—C5—C4	119.2 (3)	O3—S1—O1	112.59 (11)
C6—C5—Cl2	119.2 (3)	O3—S1—O2	113.48 (12)
C4—C5—Cl2	121.6 (3)	O1—S1—O2	111.99 (11)
C7—C6—C5	120.5 (4)	O3—S1—C1	107.50 (13)
C7—C6—H6	119.7	O1—S1—C1	104.86 (12)
C5—C6—H6	119.7	O2—S1—C1	105.69 (13)
C6—C7—C2	121.4 (3)		
			
S1—C1—C2—C7	−96.9 (3)	C5—C6—C7—C2	−0.9 (6)
S1—C1—C2—C3	81.1 (3)	C3—C2—C7—C6	1.2 (5)
C7—C2—C3—C4	0.1 (5)	C1—C2—C7—C6	179.3 (3)
C1—C2—C3—C4	−177.9 (3)	N3—C10—N1—N2	0.8 (3)
C2—C3—C4—C5	−1.8 (5)	N3—C8—N2—N1	0.4 (3)
C2—C3—C4—Cl1	178.0 (2)	C10—N1—N2—C8	−0.7 (3)
C3—C4—C5—C6	2.2 (5)	N1—C10—N3—C8	−0.5 (3)
Cl1—C4—C5—C6	−177.7 (3)	N2—C8—N3—C10	0.0 (4)
C3—C4—C5—Cl2	−177.3 (3)	C2—C1—S1—O3	48.8 (2)
Cl1—C4—C5—Cl2	2.8 (4)	C2—C1—S1—O1	168.79 (19)
C4—C5—C6—C7	−0.8 (6)	C2—C1—S1—O2	−72.7 (2)
Cl2—C5—C6—C7	178.7 (3)		

### Hydrogen-bond geometry (Å, °)

**Table:**

*D*—H···*A*	*D*—H	H···*A*	*D*···*A*	*D*—H···*A*
N1—H1M···O1^i^	0.85 (4)	1.96 (4)	2.709 (3)	146 (4)
N3—H4M···O2^ii^	0.79 (4)	2.08 (4)	2.768 (3)	146 (3)
N3—H4M···O2^iii^	0.79 (4)	2.54 (3)	3.089 (3)	128 (3)

Symmetry codes: (i) *x*, *y*+1, *z*; (ii) *x*−1, *y*, *z*; (iii) −*x*, −*y*, −*z*.
